# Frequent variations and phylogenetic relationships within the genus *Secale* identified by ND-FISH according to the genome-wide universal oligonucleotides chromosome probes

**DOI:** 10.3389/fpls.2024.1501642

**Published:** 2024-12-12

**Authors:** Zhi Li, Zixin Sun, Tianheng Ren

**Affiliations:** ^1^ State key Laboratory of Crop Gene Exploration and Utilization in Southwest China, Sichuan Agricultural University, Chengdu, China; ^2^ College of Agronomy, Sichuan Agricultural University, Chengdu, China; ^3^ Key Laboratory of Plant Genetics and Breeding at Sichuan Agricultural University of Sichuan Province, Chengdu, China

**Keywords:** rye, FISH, genetic diversity, oligonucleotides, evolution

## Abstract

**Introduction:**

Rye (*Secale cereale* L.) played a very important role in wheat genetic improvement and forage production worldwide. However, since rye is a kind of cross-pollinated plant, high levels of genetic heterozygosity and heterogeneity existed in the genome. Genome-wide variation in repeat sequences is one of the most important reasons for chromosome evolution in rye. High-precision cytological identification can effectively identify the heterochromatin or repeat sequence variations in the rye genome, and the relationship between different rye varieties can be identified while obtaining the FISH-karyotype of different rye varieties. The evolution of rye chromosomes can be analyzed by the variation degree of different probes on rye chromosomes.

**Methods:**

All materials were identified by non-denaturing fluorescence *in situ* hybridization (ND-FISH). Five probes, (AAC)_6_, Oligo-pSc119.2-1, Oligo-pTa71A-2, Oligo-pSc200, and Oligo-pSc250 were used to identify rye chromosomes.

**Results:**

15 rye varieties including *S. cereale* (cultivated rye and weedy rye), *S. strictum* (wild rye), *S. sylvestre* (wild rye), and *S. vavilovii* (wild rye) were examined by five oligonucleotides probes. 92 signal sites and 2074 signal patterns were observed, suggesting that high polymorphisms exist in the different rye genomes. The karyotypes of 15 rye varieties were obtained, the frequency of different signal types at each signal site was calculated and the model diagrams of probes (AAC)_6_, Oligo-pSc119.2-1, Oligo-pTa71A-2, Oligo-pSc200 + Oligo-pSc250 were drawn. The results showed that the rate of variation of different chromosomes of rye was not consistent. 1R, 6R, and 7R have higher variation and genetic diversity, while 2R and 3R have lower variation and are more conserved relative to other chromosomes. The results also indicated that *S. sylvestre* has a far genetic distance from other rye species, and *S. vavilovii* might be one of the ancestors of Chinese rye varieties.

**Discussion:**

Results from this study confirmed rapid chromosome change and high levels of chromosome diversity in rye.

## 1 Introduction

Rye (*Secale cereale* L, 2n=2x=14, RR genome) is a small but very important taxon in *Triticeae*. Rye is the secondary crop for food and feed, originated in the Near East, and was domesticated in Anatolia and Europe (Martis et al., 2013). Until now, Central and Eastern Europe is still the main producers of rye. Rye has great resistance to both biological and abiotic stresses and may carry a large number of disease-resistant, pest-resistant, and stress-resistant genes in the genome (Anderson et al., 2003; Li et al., 2021; Rakoczy-Trojanowska et al., 2021; Ren et al., 2022a). Therefore, rye was also used as one of the most important wheat-related species to improve the wheat genome (Ren et al., 2022a). Because rye is a cross-pollinated plant, there was a lot of gene exchange both within and between rye species, so the research on the genetic relationship between rye species has been inconsistent (Ren et al., 2011; Hagenblad et al., 2016; Guo et al., 2019; Skuza et al., 2019). However, according to different criteria, different rye species can be roughly divided into three categories, the wild species *Secale sylvestre*, the wild species *Secale strictum*, and *Secale cereale* (including cultivated and weedy rye) (Ren et al., 2011). Rye varieties were mainly cross-pollinated plants, and most of them have high genetic diversity within the species, which means there were high levels of genetic heterozygosity and heterogeneity in a rye variety (Ren et al., 2011; Bolibok-Brągoszewska et al., 2014; Guo et al., 2019). It can be seen that it is often possible to isolate different inbred lines of one rye variety by self-cross. These inbred lines from one rye variety may show different phenotypes and carry different benefit genes, thus providing different germplasm resources for the genetic improvement of wheat (Ren et al., 2022b).

There are a large number of repeated sequences in the rye genome, and the proportion of repeated sequences in the genome can reach 90% (Bauer et al., 2017). Some tandem repetitive sequences have been studied in detail based on sequence composition and genomic structure. For example, A repetition sequence of 118 bp length often appears in larger structural units, and it was named pSc119.2 (McIntyre et al., 1990). pSc200 and pSc250 have similar complex organization and contain many short direct, inverted repeats and stems (short palindromes). Therefore, for DNA containing pSc200 and pSc250, homologous recombination or chromosomal rearrangement may occur (Vershinin et al., 1995). There were many reports about the chromosome diversity within and between *triticeae* species or varieties from the results of fluorescence *in situ* hybridization (FISH) by using clones of these repetitive sequences as probes (Guo et al., 2019; Kroupin et al., 2023). Moreover, the application of oligonucleotides or multi-oligonucleotides has resulted in rapid development in the discovery of new genome- or chromosome-specific markers, such as Oligo-pSc119.2-1, Oligo-pTa71, Oligo-pSc200, and Oligo-pSc250, etc (Tang et al., 2014; Fu et al., 2015). These oligonucleotide probes have similar signal patterns to the repeat sequence probes and can be used for chromosome detection using non-denaturing fluorescence *in situ* hybridization (ND-FISH) (Tang et al., 2014; Fu et al., 2015). These oligonucleotide probes have obvious signals on the chromosomes of *triticeae* and have a very good recognition degree, which can identify different signals on chromosomes well, to quickly and accurately detect the polymorphism of signal patterns and judge the genetic differences between chromosomes of different species (Tang et al., 2014; Guo et al., 2019; Luo et al., 2022; Kroupin et al., 2023). More importantly, combining ND-FISH plus oligonucleotide probes is very cheap and much easier to operate than traditional FISH technology, making large-scale cytological identification possible.

Due to the limitation of traditional cytological markers, investigations on chromosome diversity have been limited to small numbers of individual plants (Vershinin et al., 1995; Guo et al., 2019). The research on the rye chromosomes was mainly concentrated on some cultivated rye originating from Europe and America. Genome sequencing results showed that Chinese rye (Weining) and European rye (Lo7) were very different on the genome level (Li et al., 2021; Rabanus-Wallace et al., 2021). Rye is not a major crop in China, and the relationship between Chinese rye, European rye, American rye, and wild rye is still unclear. The variation among rye varieties, as well as the evolution of rye chromosomes, remain largely unknown.

In this study, five oligonucleotide probes with high polymorphism were used with ND-FISH technology to detect the signal patterns with 15 rye varieties. High genetic diversity was observed in 15 rye varieties, and most of them have high levels of genetic heterozygosity and heterogeneity. The results also showed that *S. sylvestre* was very different from other rye, and *S. vavilovii* might be one of the ancestors of Chinese rye.

## 2 Materials and methods

### 2.1 Plant materials

Fifteen rye varieties were used in this study, and they are listed in **Table 1**. The Germplasm Resources Information Network (GRIN) of the United States Department of Agriculture (USDA) kindly provided seeds of varieties with codes beginning with PI. Our laboratory collected and kept other rye varieties (Ren et al., 2011).

**Table 1:** List of names, origin, and types for *Secale*.

Varieties	Origin	Type	Species
Weining	Southwest China	Cultivated	*S. cereale* subsp. *cereale*
Aigan	Southwest China	Cultivated	*S. cereale* subsp. *cereale*
Baili	Southwest China	Cultivated	*S. cereale* subsp. *cereale*
Jinzhou	Middle China	Cultivated	*S. cereale* subsp. *cereale*
Qinling	Northwest China	Cultivated	*S. cereale* subsp. *cereale*
Shannxi	Northwest China	Cultivated	*S. cereale* subsp. *cereale*
Chile	Chile (PI436168)	Cultivated	*S. cereale* subsp. *cereale*
AR106 BONE	America	Cultivated	*S. cereale* subsp. *cereale*
Kustro	America	Cultivated	*S. cereale* subsp. *cereale*
Segetale	Azerbaijan (PI326284)	Weedy	*S. cereale* subsp. *segetale*
Dighoricum	Russian (PI618668)	Weedy	*S. cereale subsp. dighoricum*
Vavilovii	Poland (PI618682)	Wild	*S. vavilovii*
Dalmaticum	Unknown	Wild	*S. strictum* subsp. *strictum*
Anatolicum	Canada (PI445974)	Wild	*S. strictum* subsp. *anatolicum*
Sylvestre	Ukraine (PI592294)	Wild	*S. sylvestre*

### 2.2 Chromosome identification

All materials were identified by ND-FISH. Five oligonucleotide probes, (AAC)_6_, Oligo-pSc119.2-1, Oligo-pTa71A-2, Oligo-pSc200, and Oligo-pSc250 were used to identify rye chromosomes. The sequences and the distribution of the signal patterns of the probes are listed in **Supplementary Table S1**. The details of the probes could be found in Tang et al. (2014); Cuadrado and Jouve (2002), and Luo et al. (2022). All probes were synthesized by Tsingke Biological Technology Co. Ltd. (Beijing, China). The pTa71A-2, Oligo-pSc200, and Oligo-pSc250 probes were 5’-end labeled with Cyanine Dye5 (Cy5), and the Oligo-pSc119.2-1 and (AAC)_6_ probes were 5’-end labeled with 6-carboxyfluorescein (6-FAM). Rye chromosomes were counterstained with 4’,6-diamidino-2-phenylindole (DAPI). The preparation of the experimental materials, probe labeling, *in situ* hybridization, and images captured were performed according to Tang et al. (2014) and Ren et al. (2019). Since *S. cereale* subsp. *cereale Weining* has been sequenced successfully and its mid-mitotic cytological identification results have been published, the FISH signal patterns of *S. cereale* subsp. *cereale Weining* were used as a control in this study (Li et al., 2021).

To more accurately compare the polymorphism, the signal patterns in this study were classified according to strengths, which were classified into six types: type 0 (no signals), type 1 (very weak signals), type 2 (obvious signals), type 3 (strong signals), type 4 (very strong signals) and type 5 (very strong signals and the signals radiates outside the chromosome). If there were multiple signal sites on one chromosome, these signal sites were named “probe name (chromosome-1, -2, -3, etc.)” according to the physical position from the end of the short arm to the end of the long arm. If the same chromosome of one rye variety has different probe signals, the chromosomes of this rye variety are recorded as “rye variety-1, -2, etc”.

### 2.3 Data analysis

The data of the signal types were recorded in Excel 2019. The heatmap was plotted at https://www.bioinformatics.com.cn, an online platform for data analysis and visualization (Tang et al., 2023).

## 3 Results

### 3.1 Polymorphism of the signal patterns of Oligo-pSc119.2-1

**Figure 1:**
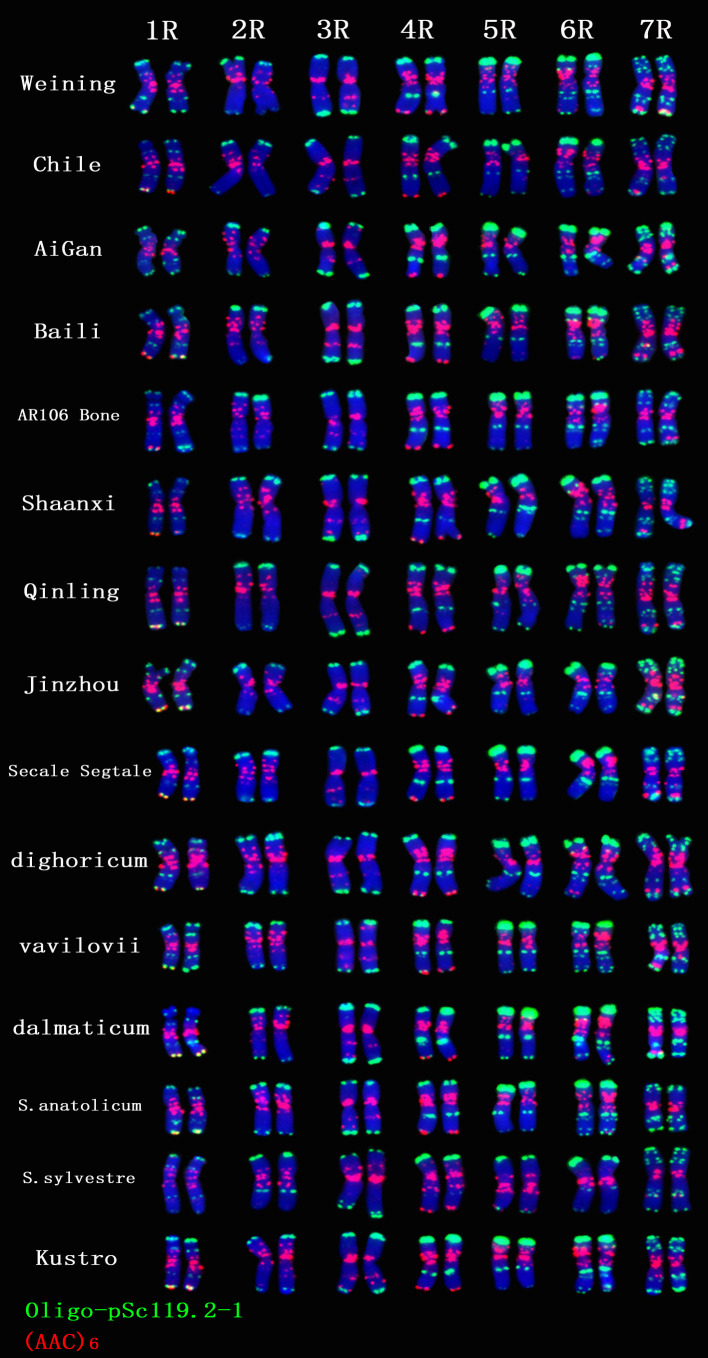
The ND-FISH results of Oligo-pSc119.2-1 and (AAC)_6_ of 1R TO 7R chromosomes for 15 rye varieties. Red signal patterns: (AAC)_6_. Green signal patterns: Oligo-pSc119.2-1. The names of rye were showed on the right.

**Figure 2:**
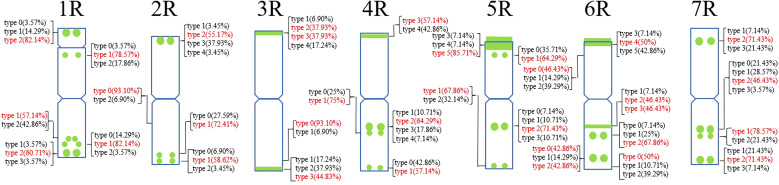
The model of the signal patterns of Oligo-pSc119.2-1. In the figure, the green spots on the chromosomes are the signal patterns at the signal sites of Oligo-pSc119.2-1 with the highest frequency. The distribution of the probe at each signal site on the chromosome and the frequency of signal intensity types were shown, the red were the types with the highest frequency at this spot.

A total of 30 Oligo-pSc119.2-1 signal sites and 748 signal patterns were shown on the chromosomes of 15 rye varieties (**Supplementary Table S2**). The Oligo-pSc119.2-1 signals showed very high polymorphism across different rye varieties. For example, different signal types of Oligo-pSc119.2-1 appeared on one signal site of 2R chromosomes of *S. cereale* subsp. *cereale Weining* (**Figure 1**). When compared with the signal patterns of *S. cereale* subsp. *cereale Weining* which was reported by Li et al. (2021), the Oligo-pSc119.2-1 signal patterns of *S. cereale* subsp. *cereale Chile* have four types of mutant at 3 signaling sites on the 1R chromosome, 2 types at 2 signaling sites on the 3R and 4R chromosome, 3 types at 3 signaling sites on the 5R chromosome, 4 types at 3 signaling sites on the 6R chromosome, and 4 types at 4 signaling sites on the 7R chromosome, respectively (**Figure 1**). All the types of signal patterns of the chromosomes of 15 rye varieties are listed in **Supplementary Table S2**. The statistics of different signal types at the same signal site were carried out, the proportion of each signal type on different signal sites was calculated (**Supplementary Table S3**), and the model of the signal patterns of Oligo-pSc119.2-1 was shown according to the types of signal patterns with the highest frequency (**Figure 2**). The distribution of the signal pattern types of different signal sites of Oligo-pSc119.2-1 on rye chromosome 1R to 7R and the frequency of different signal types were clearly shown in **Figure 2**. For example, among the five signal sites on the 1R chromosome, the most appeared signal types on Oligo-pSc119.2-1 (1RS-1), Oligo-pSc119.2-1(1RS-2), Oligo-pSc119.2-1(1RL-1), Oligo-pSc119.2-1(1RL-2), and Oligo-pSc119.2-1 (1RL-3) were type 2 (82.14%), type 1 (78.57%), type 1 (57.14%), type 1 (82.14%), and type 2 (60.71%), respectively (**Figure 2**; **Supplementary Table S3**). Compared with the signal patterns of *S. cereale* subsp. *cereale Weining* which was reported by Li et al. (2021), the mutant rates of five signal sites were higher than 60%, which were Oligo-pSc119.2-1(2RS) (62.07%), Oligo-pSc119.2-1(3RS) (62.07%), Oligo-pSc119.2-1(4RL-1) (82.14%), Oligo-pSc119.2-1(6RS-2) (60.71%), and Oligo-pSc119.2-1(7RS-2) (78.57%). The results of the mutant rates of all signal sites of Oligo-pSc119.2-1 are listed in **Supplementary Table S4**.

### 3.2 Polymorphism of the signal patterns of (AAC)_6_

**Figure 3:**
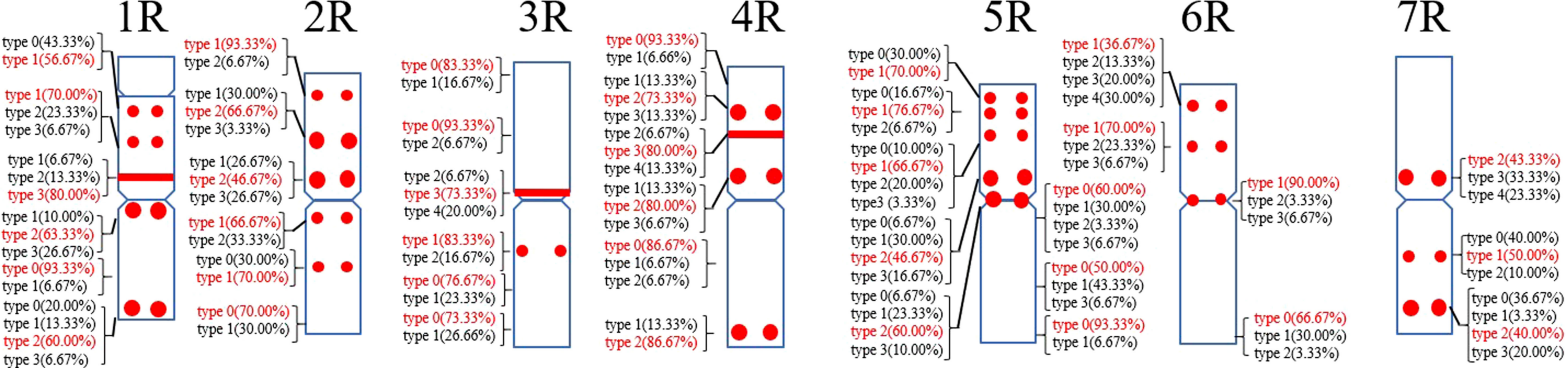
The model of the signal patterns of (AAC)_6_. In the figure, the red spots on the chromosomes are the signal patterns at the signal sites of (AAC)_6_ with the highest frequency. The distribution of the probe at each signal site on the chromosome and the frequency of signal intensity types were shown, the red were the types with the highest frequency at this spot.

A total of 39 (AAC)_6_ signal sites and 816 signal patterns were shown on the chromosomes of 15 rye varieties (**Supplementary Table S2**). As shown in **Figure 1**, the (AAC)_6_ signals showed very high polymorphism across different rye varieties. (AAC)_6_ showed obvious signal patterns on the 1R to 7R chromosomes of *S. cereale* subsp. *cereale Weining*. However, the signal patterns were not identical, and different signal types of (AAC)_6_ at different signaling sites appeared on the 2R, 3R, 4R, and 7R chromosomes (**Figure 1**; **Supplementary Table S2**). The signal patterns of (AAC)_6_ of the other 14 rye varieties also showed a lot of variations on different chromosomes. For example, when compared with the signal patterns of *S. cereale* subsp. *cereale Weining* which was reported by Li et al. (2021), the (AAC)_6_ signal patterns of *S. cereale* subsp. *cereale Chile* have 2 types of the mutant at 2 signaling sites on the 1R chromosome, 3 types at 3 signaling sites on the 2R and 4R chromosomes, 5 types at 5 signaling sites on the 5R chromosome, 2 types at 2 signaling sites on the 6R chromosome, and 1 type at 1 signaling sites on the 7R chromosome, respectively (**Figure 1**; **Supplementary Table S2**). All the types of signal patterns of (AAC)_6_ of 15 rye varieties were listed in **Supplementary Table S2**. The distribution of the signal pattern types of different signal sites of (AAC)_6_ and the frequency of different signal types are shown in **Figure 3** and **Supplementary Table S3**. For example, among the six signal sites on the 1R chromosome, the most appeared signal types on (AAC)_6_ (1R-1), (AAC)_6_ (1R-2), (AAC)_6_ (1R-3), (AAC)_6_ (1R-4), (AAC)_6_(1R-5), and (AAC)_6_ (1R-6) were type 1 (53.57%), type 1 (67.86%), type 3 (78.57%), type 2 (60.71%), type 0 (92.86%), and type 2 (57.14%), respectively (**Figure 2**; **Supplementary Table S2**). Compared with the signal patterns of *S. cereale* subsp. *cereale Weining* which was reported by Li et al. (2021), the mutant rates of seven signal sites were higher than 60%, which were (AAC)_6_3R-4 (86.21%), (AAC)_6_3R-5 (79.31%), (AAC)_6_5R-4(75%), (AAC)_6_5R-5 (82.14%), (AAC)_6_5R-6 (75%), (AAC)_6_6R-1(67.86%), and (AAC)_6_7R-3 (62.07%). The results of the mutant rates of all signal sites of (AAC)_6_ are listed in **Supplementary Table S4**.

### 3.3 Polymorphism of the signal patterns of Oligo-pSc200 + Oligo-pSc250

**Figure 4:**
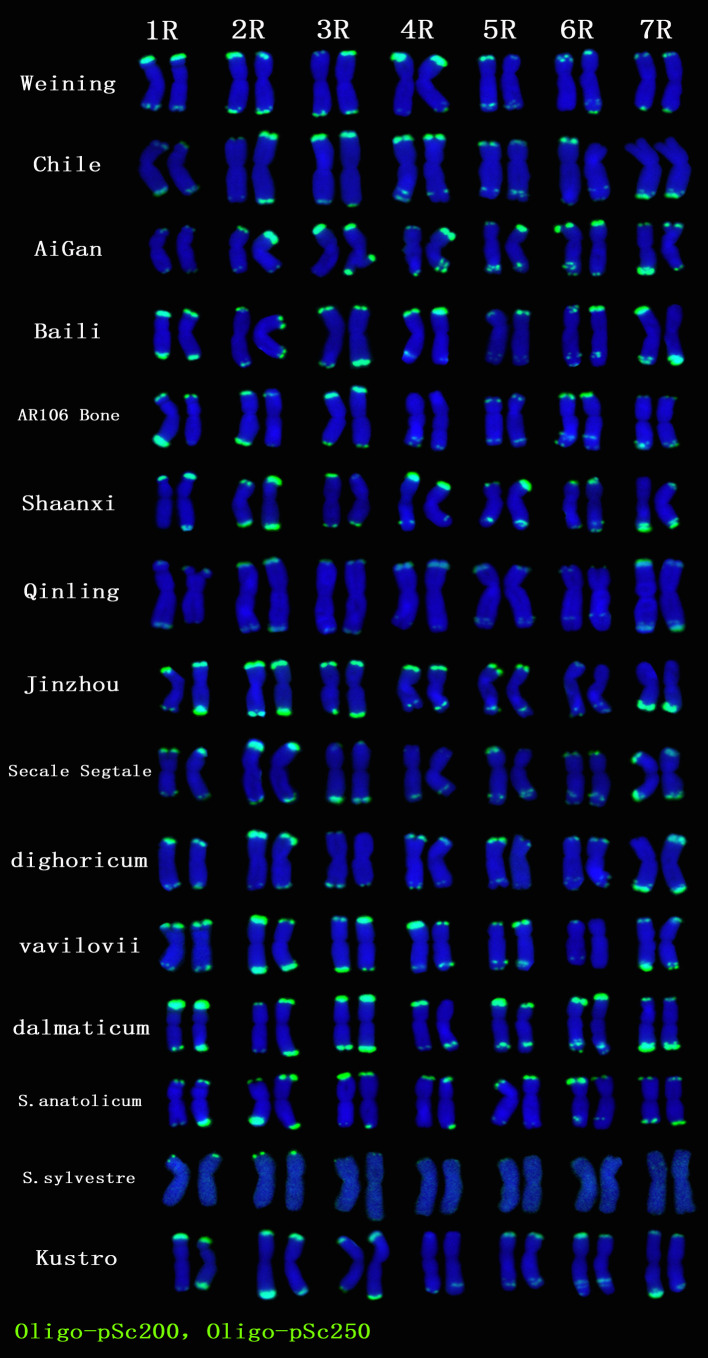
The ND-FISH results of Oligo-pSc200 + Oligo-pSc250 for 15 rye varieties. Green signal patterns: Oligo-pSc200 + Oligo-pSc250. The names of rye were showed on the right.

**Figure 5:**
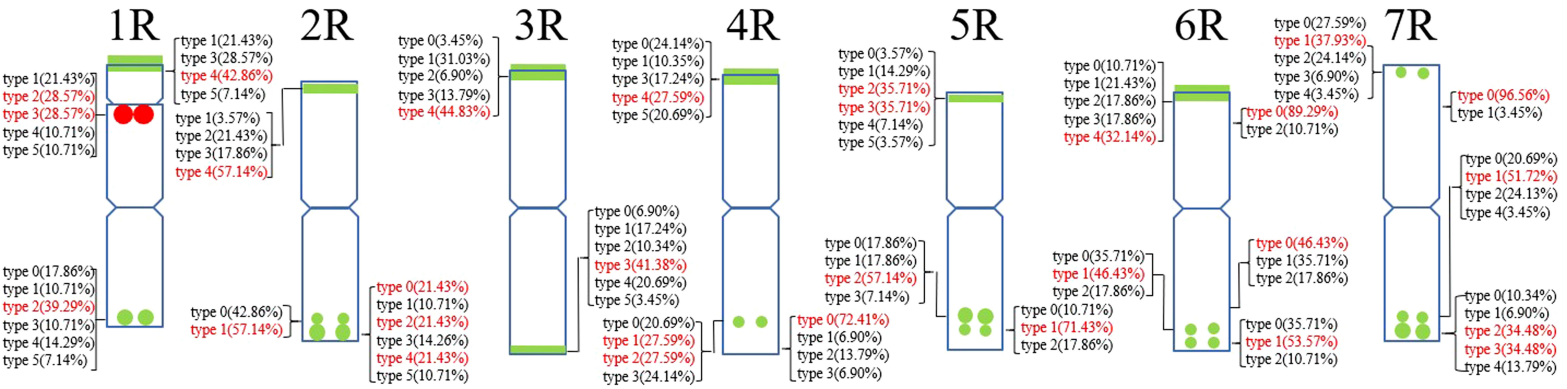
The model of the signal patterns of Oligo-pSc200 + Oligo-pSc250 and Oligo-pTa71A-2. In the figure, the red spots on the chromosomes are the signal patterns at the signal sites of Oligo-pTa71A-2 with the highest frequency. The green spots on the chromosomes are the signal patterns at the signal sites of Oligo-pSc200 + Oligo-pSc250 with the highest frequency. The distribution of the probe at each signal site on the chromosome and the frequency of signal intensity types were shown. The red were the types with the highest frequency at this spot.

A total of 22 Oligo-pSc200 + Oligo-pSc250 signal sites and 480 signal patterns were shown on the chromosomes of 15 rye varieties (**Supplementary Table S2**). As shown in **Figure 4**, the Oligo-pSc200 + Oligo-pSc250 signals showed very high polymorphism across different rye varieties, as well as the signal patterns of (AAC)_6_ and Oligo-pSc119.2-1. The signal patterns of Oligo-pSc200 + Oligo-pSc250 of *S. cereale* subsp. *cereale Weining* were different at the different signal sites on the 3R, 4R, and 7R chromosomes (**Figure 4**; **Supplementary Table S2**). The signal patterns of Oligo-pSc200 + Oligo-pSc250 of the other 14 rye varieties also showed a lot of variations on different chromosomes. For example, when compared with the signal patterns of *S. cereale* subsp. *cereale Weining* which was reported by Li et al. (2021), the Oligo-pSc200 + Oligo-pSc250 signal patterns of *S. cereale* subsp. *cereale Chile* have 3 types of the mutant at 2 signaling sites on the 1R chromosome, 2 types at 2 signaling sites on the 2R chromosome, 1 type at 1 signaling sites on the 3R chromosome, 3 types at 3 signaling sites on the 4R chromosome, 1 type at 1 signaling site on the 5R chromosome, 4 types at 4 signaling sites on the 6R chromosome, and 2 types at 2 signaling sites on the 7R chromosome (**Supplementary Table S2**; **Figure 4**). The signal patterns of Oligo-pSc200 + Oligo-pSc250 of *S*. *sylvestre* were completely different from other rye varieties. Most of the signal patterns of Oligo-pSc200 + Oligo-pSc250 were disappeared or very weak in *S. sylvestre* (**Figure 4**). All the types of signal patterns of Oligo-pSc200 + Oligo-pSc250 of 15 rye varieties were listed in **Supplementary Table S2**. The distribution of the signal pattern types of different signal sites of Oligo-pSc200 + Oligo-pSc250 and the frequency of different signal types are shown in **Figure 5** and **Supplementary Table S3**. For example, among the two signal sites on the 1R chromosome, the most appeared signal types on Oligo-pSc200+Oligo-pSc250(1RS) and Oligo-pSc200+Oligo-pSc250(1RL) were type 4 (42.86%) and type 2 (39.29%), respectively (**Figure 5**; **Supplementary Table S3**). Compared with the signal patterns of *S. cereale* subsp. *cereale Weining* which was reported by Li et al. (2021), the mutant rates of 10 signal sites were higher than 60%, which were Oligo-pSc200 + Oligo-pSc250(1RL) (60.71%), Oligo-pSc200 + Oligo-pSc250(2RL-2) (85.71%), Oligo-pSc200 + Oligo-pSc250(4RS) (79.31%), Oligo-pSc200 + Oligo-pSc250(4RL-1) (72.41%), Oligo-pSc200 + Oligo-pSc250(5RS) (64.29%), Oligo-pSc200 + Oligo-pSc250(6RS-1) (82.14%), Oligo-pSc200 + Oligo-pSc250(6RL-1) (64.29%), Oligo-pSc200 + Oligo-pSc250(6RL-3) (89.29%), Oligo-pSc200 + Oligo-pSc250(7RL-1) (79.31%), and Oligo-pSc200 + Oligo-pSc250(7RL-2) (65.52%). The results of the mutant rates of all signal sites of Oligo-pSc200 + Oligo-pSc250 are listed in **Supplementary Table S4**.

### 3.4 Polymorphism of the signal patterns of Oligo-pTa71A-2

**Figure 6:**
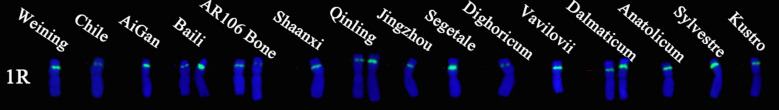
The ND-FISH results of Oligo-pTa71A-2 for 15 rye varieties. The names of rye were showed on the top of the chromosomes. Green: Oligo-pTa71A-2.

Oligo-pTa71A-2 has one signal site at the junction of the 1R short arm and the satellite, that is, the nucleolus organizer region. A total of 30 signal patterns were shown on the chromosomes of 15 rye varieties. In *S. cereale* subsp. *cereale Weining*, there was only one type of signal pattern of Oligo-pTa71A-2 (type 3). However, as shown in **Figure 6**, the Oligo-pTa71A-2 signals also showed high polymorphism across different rye varieties when they were compared with *S. cereale* subsp. *cereale Weining*. For example, the Oligo-pTa71A-2 signal patterns on the 1R chromosome of *S. cereale* subsp. *cereale Chile* (type 1) and *S. cereale* subsp. *Dighoricum* (type 1) were significantly weaker, while the signal patterns on the 1R chromosome of *S. cereale* subsp. *Segetale* (type 5) and *S. sylvestre* (type 4) were significantly stronger (**Supplementary Table S4**; **Figure 6**). The signal patterns of other rye varieties also showed many different types of variation, and the signal types of Oligo-pTa71A-2 of all 15 rye varieties were listed in **Supplementary Table S2**. The frequency of different signal types of Oligo-pTa71A-2 was type 1 (21.43%), type 2 (28.57%), type 3 (28.57), type 4 (10.71), and type 5 (10.71%), respectively (**Figure 5**; **Supplementary Table S3**).

### 3.5 The variation in different chromosomes

Each chromosome showed a high level of genetic diversity. However, the degree of polymorphism of different probes on different chromosomes is different. Compared with the signal patterns of *S. cereale* subsp. *cereale Weining* which was reported by Li et al. (2021), the mutant rates based on all signal patterns of the chromosomes from high to low were: 6R (47.11%), 7R (43.94%), 5R (39%), 1R (35.48%), 4R (33.85%), 2R (28%), and 3R (25.15%) on average (**Supplementary Table S4**); based on the signal patterns of Oligo-pSc119.2-1 of the chromosomes from high to low were: 6R (48.33%), 4R (45%), 7R(44.17%), 3R(40%), 1R (28.67%), 5R (26%), and 2R (20%) on average (**Supplementary Table S4**); based on the signal patterns of (AAC)_6_ of the chromosomes from high to low were: 5R (45%), 7R (42.22%), 6R (34.17%), 1R (29.45%), 2R (22%), 4R(15.56%), and 3R (12.78) on average (**Supplementary Table S4**); based on the signal patterns of Oligo-pSc200 + Oligo-pSc250 of the chromosomes from high to low were: 6R (56%), 4R (55.56%), 1R (55%), 2R (53%), 7R (45%), 5R (42%), and 3R (40%) on average (**Supplementary Table S4**). Based on the signal patterns of Oligo-pTa71A-2 of the 1R chromosomes, the mutant rate of the signal patterns of Oligo-pTa71A-2 is as high as 71.43% (**Supplementary Table S4**).

### 3.6 Cluster analysis

**Figure 7:**
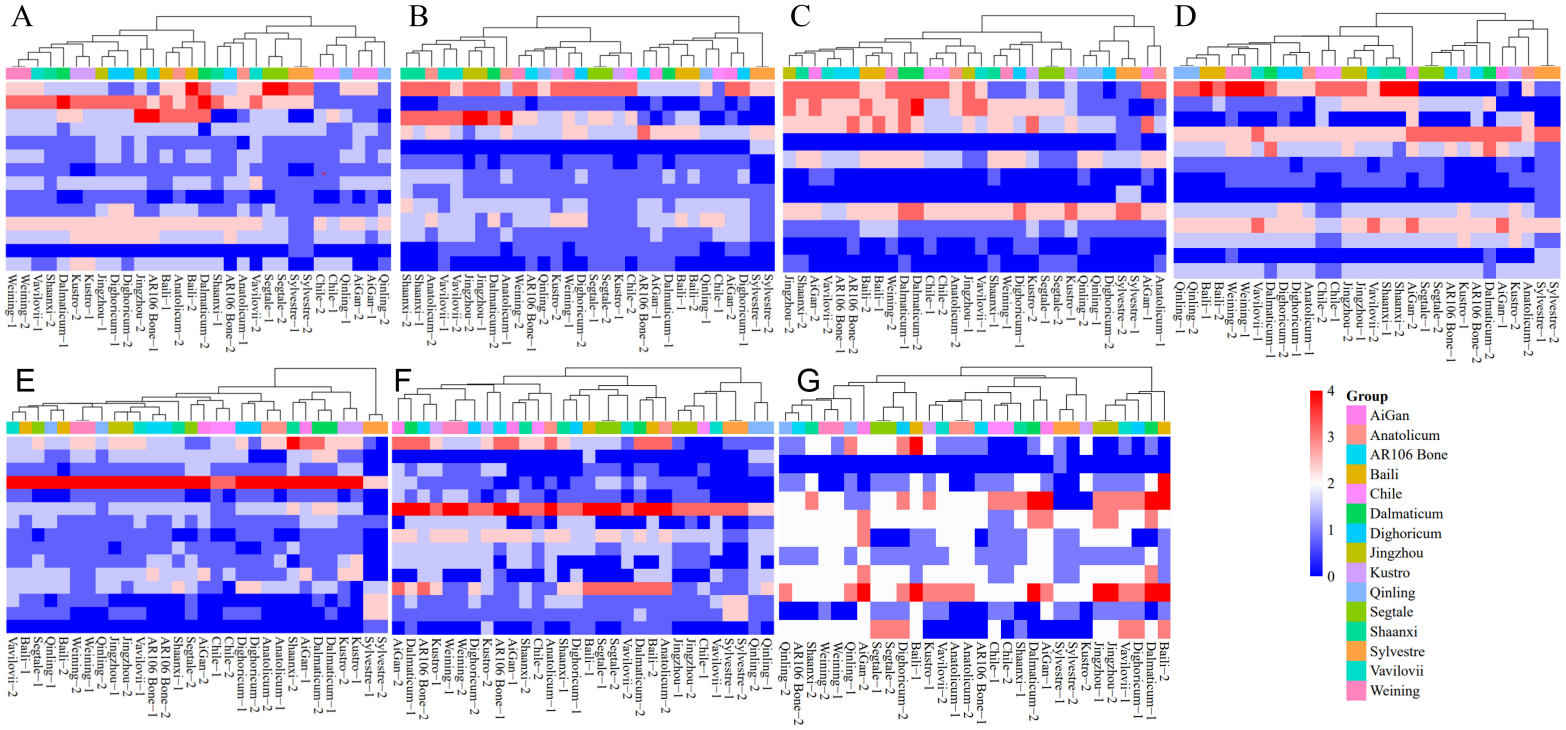
Heatmap clustering analysis of 1R to 7R chromosomes of 15 rye varieties. **(A)** Heatmap clustering analysis of 1R chromosome. **(B)** Heatmap clustering analysis of 2R chromosome. **(C)** Heatmap clustering analysis of 3R chromosome. **(D)** Heatmap clustering analysis of 4R chromosome. **(E)** Heatmap clustering analysis of 5R chromosome. **(F)** Heatmap clustering analysis of 6R chromosome. **(G)** Heatmap clustering analysis of 7R chromosome. All signal patterns showed on the chromosomes were used for analysis.

The heat maps based on signal types of different chromosomes of different rye further illustrate the evolutionary relationship between different chromosomes of different rye varieties (**Figure 7**). For example, the 2R, 4R, and 5R chromosomes of *S*. *sylvestre* were divided into different clusters from other rye. On the other hand, the 1R, 3R, and 7R chromosomes of S. *sylvestre* were closely related to another wild rye *S. strictum* subsp. *Anatolicum*. Moreover, the 1R, 3R, and 6R of *S. sylvestre* were also closely related to *S. cereale* subsp. *Segetale*, Dighoricum-2 (*S. cereale* subsp. *Dighoricum*), and Vavilovii-1 (*S. vavilovii*), respectively (**Figure 7**).

The results showed that the 1R chromosomes of different rye had significant differences, and all 15 rye varieties could be classified into 4 clusters (**Figure 7A**). Clusters 1 and 2 are more closely related, while clusters 3 and 4 are more closely related. In cluster 1, *S. cereale* subsp. *cereale Qinling*, *S. cereale* subsp. *cereale Aigan*, and *S. cereale* subsp. *cereale Chile* were included. In cluster 2, *S. cereale* subsp. *Segetale*, *S. sylvestre*, Vavilovii-2 (*S. vavilovii*), Shaanxi-1 (*S. cereale* subsp. *cereale Shaanxi*), AR106 BONE-2 (*S. cereale* subsp. *cereale AR106BONE*), and Anatonicum-1 (*S. strictum* subsp. *Anatolicum*) were included. In cluster 3, *S. cereale* subsp. *cereale Baili*, Anatonicum-2 (*S. strictum* subsp. *Anatolicum*), and Dalmaticum-2 (*S. strictum* subsp. *strictum Dalmaticum*) were included. In cluster 4, *S. cereale* subsp. *cereale Weining*, Vavilovii-1 (*S. vavilovii*), Shannxi-2 (*S. cereale* subsp. *cereale Shaanxi*), Dalmaticum-1 (*S. strictum* subsp. *strictum Dalmaticum*), *S. cereale* subsp. *cereale Kustro*, *S. cereale* subsp. *cereale Jingzhou*, *S. cereale* subsp. *Dighoricum*, and AR106 BONE-1 (*S. cereale* subsp. *cereale AR106BONE*) were included. *S. vavilovii* had a closer relationship with *S. cereale* subsp. *cereale Weining*, *S. cereale* subsp. *cereale Shaanxi*, and *S. strictum* subsp. *Anatolicum.*

The 2R chromosomes of different rye varieties also had significant differences, and all 15 rye varieties could be classified into 4 clusters (**Figure 7B**). Clusters 1 and 2 are more closely related, while clusters 3 and 4 are more closely related. In cluster 1, only *S. sylvestre* was included. In cluster 2, AR106 Bone-2 (*S. cereale* subsp. *cereale AR106BONE*), *S. cereale* subsp. *cereale Aigan*, *S. cereale* subsp. *cereale Baili*, Dalmaticum-1 (*S. strictum* subsp. *strictum Dalmaticum*), Qinling-1 (*S. cereale* subsp. *cereale Qinling*), Dighoricum-1 (*S. cereale* subsp. *Dighoricum*), and Chile-1 (*S. cereale* subsp. *cereale Chile*) were included. In cluster 3, *S. cereale* subsp. *cereale Weining*, AR106 BONE-1 (*S. cereale* subsp. *cereale AR106BONE*), Qinling-2 (*S. cereale* subsp. *cereale Qinling*), *S. cereale* subsp. *cereale Kustro*, *S. cereale* subsp. *Segetale*, Chile-2 (*S. cereale* subsp. *cereale Chile*), Dighoricum-2 (*S. cereale* subsp. *Dighoricum*) were included. In cluster 4, *S. cereale* subsp. *cereale Shaanxi*, *S. strictum* subsp. *Anatolicum*, *S. vavilovii*, *S. cereale* subsp. *cereale Jingzhou*, and Dalmaticum-2 (*S. strictum* subsp. *strictum Dalmaticum*) were included. *S. vavilovii* had a much closer relationship with *S. cereale* subsp. *cereale Shaanxi*, and *S. strictum* subsp. *Anatolicum*.

The 3R chromosomes also showed high differences among different rye, and 15 rye varieties could be classified into 4 clusters, and clusters 2, 3, and 4, are more closely related (**Figure 7C**). In cluster 1, *S. cereale* subsp. *cereale Qinling*, Dighoricum-2 (*S. cereale* subsp. *Dighoricum*), *S. sylvestre*, Aigan-1 (*S. cereale* subsp. *cereale Aigan*) and Anatolicum-1 (*S. strictum* subsp. *Anatolicum*) were included. In cluster 2, *S. cereale* subsp. *Segetale*, *S. cereale* subsp. *cereale Kustro*, Dighoricum-1 (*S. cereale* subsp. *Dighoricum*), Weining-1 (*S. cereale* subsp. *cereale Weining*), and Shaanxi-1 (*S. cereale* subsp. *cereale Shaanxi*) were included. In cluster 3, *S. cereale* subsp. *cereale Chile*, Anatolicum-2 (*S. strictum* subsp. *Anatolicum*), Jingzhou-1 (*S. cereale* subsp. *cereale Jingzhou*), and Vavilovii-1 (*S. vavilovii*) were included. In cluster 4, Jinzhou-2 (*S. cereale* subsp. *cereale Jingzhou*), Shaanxi-2 (*S. cereale* subsp. *cereale Shaanxi*), Aigan-2 (*S. cereale* subsp. *cereale Aigan*), Vavilovii-2 (*S. vavilovii*), *S. cereale* subsp. *cereale AR106BONE*, *S. cereale* subsp. *cereale Baili*, Weining-2 (*S. cereale* subsp. *cereale Weining*), and *S. strictum* subsp. *strictum Dalmaticum* were included. *S. vavilovii* had a much closer relationship with *S. cereale* subsp. *cereale Jingzhou*, *S. cereale* subsp. *cereale Shaanxi*, *S. cereale* subsp. *cereale Aigan*, and *S. cereale* subsp. *cereale AR106BONE*.

The 4R chromosomes showed higher differences among different rye, and 15 rye varieties could be classified into 7 clusters. Clusters 1, 2, and 3 are more closely related, while clusters 4, 5, 6, and 7 are more closely related (**Figure 7D**). In cluster 1, only *S. sylvestre* was included. In cluster 2, Aigan-1 (*S. cereale* subsp. *cereale Aigan*), Kustro-1 (*S. cereale* subsp. *cereale Kustro*), and Anatolicum-2 (*S. strictum* subsp. *Anatolicum*) were included. In cluster 3, *S. cereale* subsp. *Segetale*, *S. cereale* subsp. *cereale AR106BONE*, Kustro-1 (*S. cereale* subsp. *cereale Kustro*), and Dalmaticum-2 (*S. strictum* subsp. *strictum Dalmaticum*) were included. In cluster 4, only Aigan-2 (*S. cereale* subsp. *cereale Aigan*) was included. In cluster 5, *S. cereale* subsp. *cereale Jingzhou*, *S. cereale* subsp. *cereale Shaanxi*, and *S. vavilovii* were included. In cluster 5, only *S. cereale* subsp. *cereale Chile* was included. In cluster 6, Anatolicum-1 (*S. strictum* subsp. *Anatolicum*) and *S. cereale* subsp. *Dighoricum* were included. In cluster 7, *S. cereale* subsp. *cereale Qinling*, *S. cereale* subsp. *cereale Baili*, *S. cereale* subsp. *cereale Weining*, Dalmaticum-1 (*S. strictum* subsp. *strictum Dalmaticum*), and *S. vavilovii* were included. *S. vavilovii* had a closer relationship with Chinese rye landraces and wild rye.

Based on the signal patterns of 5R, 15 rye varieties could be classified into 5 clusters, and clusters 3, 4, and 5 are more closely related (**Figure 7E**). In cluster 1, only *S. sylvestre* was included. In cluster 2, Shaanxi-2 (*S. cereale* subsp. *cereale Shaanxi*), Aigan-1 (*S. cereale* subsp. *cereale Aigan*), Dalmaticum (*S. strictum* subsp. *strictum Dalmaticum*), and Kustro (*S. cereale* subsp. *cereale Kustro*) were included. In cluster 3, *S. strictum* subsp. *Anatolicum* and *S. cereale* subsp. *Dighoricum* were included. In cluster 4, Chile (*S. cereale* subsp. *cereale Chile*), Aigan-2 (*S. cereale* subsp. *cereale Aigan*), and Segetale-2 (*S. cereale* subsp. *Segetale*) were included. Cluster 5 could be divided into 4 sub-clusters. In sub-cluster 1, only Shaanxi-1 (*S. cereale* subsp. *cereale Shaanxi*) was included. In sub-cluster 2, AR106 BONE (*S. cereale* subsp. *cereale AR106BONE*), Vavilovii-1 (*S. vavilovii*), and *S. cereale* subsp. *cereale Jingzhou* were included. In sub-cluster 3, *S. cereale* subsp. *cereale Weining* and Qinling-2 (*S. cereale* subsp. *cereale Qinling*) were included. In sub-cluster 4, Vavilovii-2 (*S. vavilovii*), *S. cereale* subsp. *cereale Baili*, Segetale-1 (*S. cereale* subsp. *Segetale*), and Qinling-1 (*S. cereale* subsp. *cereale Qinling*) were included. *S. vavilovii* had a closer relationship with two Chinese rye *S. cereale* subsp. *cereale Baili* and *S. cereale* subsp. *cereale Jingzhou*.

Based on the signal patterns of 6R, 15 rye varieties could be classified into 3 clusters, and clusters 2 and 3 are more closely related (**Figure 7F**). In cluster 1, *S. cereale* subsp. *cereale Qinling*, *S. sylvestre*, Vavilovii-1 (*S. vavilovii*), *S. cereale* subsp. *cereale Jingzhou*, and Chile-1 (*S. cereale* subsp. *cereale Chile*) were included. Cluster 2 could be divided into 2 sub-clusters. In sub-cluster 1, Baili-2 (*S. cereale* subsp. *cereale Baili*), Anatolicum-2 (*S. strictum* subsp. *Anatolicum*), and Dalmaticum-2 (*S. strictum* subsp. *strictum Dalmaticum*) were included. In sub-cluster 2, Vavilovii-2 (*S. vavilovii*), *S. cereale* subsp. *Segetale*, Baili-1 (*S. cereale* subsp. *cereale Baili*), Dighoricum-1 (*S. cereale* subsp. *Dighoricum*), and Shaanxi-1 (*S. cereale* subsp. *cereale Shaanxi*) were included. Cluster 3 could be divided into 3 sub-clusters. In sub-cluster 1, Anatolicum-1 (*S. strictum* subsp. *Anatolicum*), Chile-2 (*S. cereale* subsp. *cereale Chile*), Shaanxi-2 (*S. cereale* subsp. *cereale Shaanxi*), and Aigan-1(*S. cereale* subsp. *cereale Aigan*) were included. In sub-cluster 2, Kustro-2 (*S. cereale* subsp. *cereale Kustro*) and AR106 BONE-1 (*S. cereale* subsp. *cereale AR106BONE*) were included. In sub-cluster 3, Aigan-2 (*S. cereale* subsp. *cereale Aigan*), Dalmaticum-1 (*S. strictum* subsp. *strictum Dalmaticum*), AR106 BONE-2 (*S. cereale* subsp. *cereale AR106BONE*), Kustro-1 (*S. cereale* subsp. *cereale Kustro*), *S. cereale* subsp. *cereale Weining*, and Dighoricum-2 (*S. cereale* subsp. *Dighoricum*), were included. *S. vavilovii* had a closer relationship with three Chinese rye *S. cereale* subsp. *cereale Qinling*, *S. cereale* subsp. *cereale Jingzhou*, and *S. cereale* subsp. *cereale Baili*.

Based on the signal patterns of 7R, 15 rye varieties could be classified into 3 clusters, and clusters 2 and 3 are more closely related (**Figure 7G**). In cluster 1, Vavilovii-1 (*S. vavilovii*), Dighoricum-1 (*S. cereale* subsp. *Dighoricum*), Dalmaticum-1 (*S. strictum* subsp. *strictum Dalmaticum*), Baili-2 (*S. cereale* subsp. *cereale Baili*), and *S. cereale* subsp. *cereale Jingzhou* were included. Cluster 2 could be divided into 4 sub-clusters. In sub-cluster 1, *S. sylvestre* and *S. cereale* subsp. *cereale Kustro* were included. In sub-cluster 2, Aigan-2 (*S. cereale* subsp. *cereale Aigan*) and Dalmaticum-2 (*S. strictum* subsp. *strictum Dalmaticum*) were included. In sub-cluster 3, *S. cereale* subsp. *cereale Chile* and Shaanxi-1 (*S. cereale* subsp. *cereale Shaanxi*) were included. In sub-cluster 4, Vavilovii-2 (*S. vavilovii*), *S. strictum* subsp. *Anatolicum*, AR106 BONE-1 (*S. cereale* subsp. *cereale AR106BONE*), and Kustro-1 (*S. cereale* subsp. *cereale Kustro*) were included. Cluster 3 could be divided into 2 sub-clusters. In sub-cluster 1, *S. cereale* subsp. *Segetale*, Dighoricum-2 (*S. cereale* subsp. *Dighoricum*), and Baili-1 (*S. cereale* subsp. *cereale Baili*) were included. In sub-cluster 2, *S. cereale* subsp. *cereale Qinling*, *S. cereale* subsp. *cereale Weining*, Shaanxi-2 (*S. cereale* subsp. *cereale Shaanxi*), Aigan-2 (*S. cereale* subsp. *cereale Aigan*), and *S. cereale* subsp. *cereale AR106BONE* were included. *S. vavilovii* had a closer relationship with *S. cereale* subsp. *cereale Jingzhou*.

## 4 Discussion

### 4.1 Chromosome identified by FISH technique

Cytogenetics enables the simultaneous of the genome and comparison of the chromosomes between different species. Therefore, cytogenetics has been widely used in wheat improvement programs (Gill, 2022; Kroupin et al., 2023). As one of the most valuable tools of cytogenetics, FISH is a powerful tool that enables to performance of systematic, evolutionary, and population studies of wheat wild relatives as well as to characterize alien introgression into the wheat genome (Kroupin et al., 2023). Triticeae probes based on satellite repeats have been widely used for chromosome analysis, and many new probes, especially for oligonucleotide and multi-oligonucleotides were developed and were quickly used in chromosome identification. For example, to verify the distribution of satellite DNA in *Thinopyrum bessarabicum* chromosomes, new oligonucleotides were designed based on the sequences of satellite clusters or the coding sequences of 5S rDNA. These probes can distinguish all wheat and *Th. bessarabicum* chromosomes after one round of FISH (Chen et al., 2019). The genetic polymorphisms among different *Dasypyrum villosum* accessions were also determined by cytological analysis. One multi-oligonucleotides probe ONPM4# (contained six oligonucleotides: pAs1-1, pAs1-3, pAs1-4, pAs1-6, AFA-3, and AFA-4), and two oligonucleotides probes (GAA)_10_ and pSc119.2-1 were used, and a total of 106 polymorphic chromosomes were identified (Wu et al., 2023). The chromosomes of *Agropyron cristatum* were identified by five tandem repeat probes, in addition to 5S and 45S ribosomal DNA and rye sub-telomeric repeats pSc119.2 and pSc200. As a result, structural rearrangements were observed for chromosomes 2P, 4P, 5P, 6P, and 7P of *A. cristatum*, and chromosomal inversions were also found for the pericentric region of 4P and whole chromosome arm 6PL (Said et al., 2018). Chromosome-specific FISH markers for *Psathyrostachys huashanica* were developed by Zhang et al. (2022). The results indicated that the combination of pSc200, pTa71A-2, and Oligo-44 can distinguish all Ns chromosomes from wheat chromosomes in the wheat background. Structural chromosome variations (SCVs) are large-scale genomic variations that can be also detected by FISH (Zhao et al., 2022; Ren et al., 2016). For example, 543 wheat accessions from China were identified by oligonucleotide probe multiplex FISH, and 139 SCVs including translocations, pericentric inversions, presence/absence variations, and copy number variations were identified at 230 loci (Zhao et al., 2022). A complex chromosome rearrangement line with balanced reciprocal translocations 1RS.3BL and 3BS.1BL, and a complex chromosome translocation line with 3DS.4BS^DS^ and 3DL-4BS^PS^.4BL was also identified by FISH (Ren et al., 2016; Li et al., 2022). Ren et al. (2017) found mutants on the 4A and 5A chromosomes of wheat in a newly developed 1RS.1BL translocation line when compared with the wheat parent. Five different oligonucleotide probes (Oligo-pSc119.2-1, Oligo-pTa535-1, Oligo-Ku, Oligo-pSc200, and Oligo-pSc250) were used with ND-FISH to examine 21 wheat cultivars, and 17.6% of the A-genome chromosomes, 25.9% of the B-genome chromosomes, and 8.9% of the D-genome chromosomes showed obvious mutations when they were compared to the standard signal patterns (Ren et al., 2019). The results of cytological identification can also shed light on some related theories such as recombination, species evolution, and gene functions. For example, to precisely identify *Triticum timopheevii* chromosomes and to trace the evolution of *Triticum zhukovskyi*, several probes, such as pSc119.2, pTa71, pAs1, pTa535-1, (GAA)_9_, and (CTT)_10_ were used. As a result, the origin of *T. zhukovskyi* from the hybridization of *T. timopheevii* with *T. monococcum* was confirmed (Badaeva et al., 2016). To study the effects of structural variations of chromosomes during the meiotic recombination, the wheat lines with different 5A structures were used to investigate their meiotic recombination by ND-FISH. The results showed that the smaller structural difference between the 5A in the distal regions resulted in a higher recombination frequency in the interstitial region (Zou et al., 2022). ND-FISH with oligonucleotide probes derived from tandem repeats and single-copy FISH were used to investigate recombination in three kinds of 5AL, and the variations of the signal patterns of Oligo-pSc119.2-1, Oligo-pTa535–1, Oligo-713, Oligo-275.1, Oligo-18, and the variations of the signal patterns of the single-copy FISH probes showed the structural variations caused by tandem repeats might be one of the factors affecting meiotic recombination in wheat (Zou et al., 2021). The FISH results of the localization of CENH3 in the centromere of soybean, emphasize the role of centromere satellites in maintaining stable positions, underscoring their importance in centromere organization (Liu et al., 2023).

As one of the most valuable wheat-related species, the precise identification of the rye chromosomes and precise recognition of the rye chromosomes in the wheat genetic background were important goals for wheat distance hybridization breeding programs. Several FISH probes were developed based on the repetitive sequences, such as pSc119.2, pAs1, pTa-535, pTa71, CCS1, and PAWRC.1 (Tang et al., 2014). These probes can distinguish wheat and rye chromosomes in one cell and play important roles in wheat-rye cytogenetics identification studies (An et al., 2019; Ren et al., 2017, Ren et al., 2022a; Ren et al., 2022b; Han et al., 2020, 2023; Kroupin et al., 2023). Several new oligonucleotide probes that can be used with ND-FISH were developed in recent years and quickly used in rye chromosome identification due to their significant advantages, such as being more convenient, precise, and cheap (Tang et al., 2014; Fu et al., 2015). For example, six rye cultivars were analyzed by using oligonucleotides TAMRA-oligo-6 and FAM-pSc119.2-1 to represent the signal patterns of pSc200 and pSc119.2, and 73 types of heterochromatin blocks were identified on all seven chromosomes (Guo et al., 2019).

In this study, seven oligonucleotide probes were employed to identify the genetic polymorphisms among different rye varieties. In previous studies, these probes have been proven can replace the roles of repetitive sequences pAs1, pSc119.2, pTa-535, pTa71, and rye genomic DNA in FISH analysis of wheat, rye, and hybrids derived from wheat × rye by DN-FISH (Tang et al., 2014; Fu et al., 2015; Ren et al., 2019; Ren et al., 2022a; Ren et al., 2022b; Luo et al., 2022). The results showed that the oligonucleotide probes Oligo-pSc119.2-1, Oligo-pTa71A-2, Oligo-pSc200 + Oligo-pSc250, and (AAC)_6_ produced high-resolution signal patterns not only showed different signal patterns in the rye chromosomes but also revealed the varied distribution of these probes among chromosomes and varieties (**Figures 1**, **4**, **6**). A total of 92 polymorphic signal patterns were identified from 15 rye varieties and high levels of chromosomal heterozygosity and heterogeneity were observed. The results showed that these oligonucleotide probes could effectively detect the differences in the signal patterns in rye chromosomes, and the polymorphisms of the chromosomes could be easily observed according to these probes by the ND-FISH method. Compared with the signal patterns of *S. cereale* subsp. *cereale Werining*, which was reported by Li et al. (2021), the total mutant rates of the other 14 rye varieties ranged from 25.15% to 47.11% (**Supplementary Table S4**). The results suggested that 6R and 7R have the highest variation and the genetic diversity, while 2R and 3R have the lowest variation and are more conserved relative to other chromosomes. Oligo-pTa71A-2 had the highest variation rate among all probes, suggesting that the distal of the 1RS chromosome may have more variation and higher genetic diversity (Luo et al., 2022).

### 4.2 The genetic relationship between different rye varieties

The phylogenetic relationships and taxonomy within the genus *Secale* have long been the subject of controversy (Ren et al., 2011; Skuza et al., 2019). In the beginning, according to the results of the morphological, ecological, and earlier cytological investigations, scientists believed that *S. vavilovii* should be an ancestor of the cultivated rye (Vavilov, 1926). Several useful genes, such as self-fertility, high protein content, resistance to diseases, and resistance to sprouting were successfully used for the improvement of cultivated rye in Europe (Meier et al., 1996). The analyses of RFLP of mitochondrial DNA (mtDNA) indicated that *S. vavilovii* had a close genetic relationship with *S. strictum* and *S. cereal*e, but a far genetic relationship with *S. sylvestre* and *S. cereale* subsp. *Segetale* (Skuza et al., 2007). The analysis of ISSR also indicated that the genetic distance of *S. vavilovii* was closer to *S. cereale* but far from *S. sylvestre* and *S. strictum* subsp. *Anatolicum* (Ren et al., 2011). Based on the analysis of noncoding regions of the chloroplast (cpDNA) and mtDNA, the results indicated that *S. Vavilovii* was very similar to *S. cereale* (Skuza et al., 2019). Schreiber et al. (2019) found that there was only weak genetic differentiation between *S. vavilovii* and domesticated rye with likely gene flow. All the results indicated that *S. vavilovii* has a very close genetic relationship with cultivated rye, which supported the assumption that *S. vavilovii* might share a common ancestor with cultivated rye, or *S. vavilovii* was one of the ancestors of cultivated rye (Skuza et al., 2019). China has no history of growing and domesticating rye. Therefore, these undomesticated or artificially selected rye in China tend to have richer genetic resources. In recent years, a large number of disease-resistance genes have been found in these Chinese rye varieties and used in wheat breeding (Ren et al., 2017; Ren et al., 2022a; Ren et al., 2022b). The previous molecular analysis suggested that rye had been introduced from its origin center to northwest China and then spread to southwest China (Ren et al., 2011). During the spread process, genetic differentiation likely occurred. There were big differences between the genomes of Chinese rye (Weining) and European rye (Lo7) (Rabanus-Wallace et al., 2021; Li et al., 2021). The rye varieties from northwestern China were more genetically similar to the rye varieties of western countries than the rye varieties from southwestern China (Ren et al., 2011). In this study, based on the signal patterns of different probes, it looked like the Chinese rye had a closer genetic relationship with *S. vavilovii* (**Figure 7**), which suggested that *S. vavilovii* might be one of th*e* ancestors of Chinese rye.

*S. sylvestre* was another wild rye and had a far genetic distance from other rye varieties. The lowest call rates were observed on the Rye600k array in samples from *S. vavilovii* (87.5%) and *S. sylvestre* (84.3%), consistent with their evolutionary distance from cultivated rye (Bauer et al., 2017). Based on the results characteristics of internal transcribed spacer (ITS) rDNA sequences suggested that *S. sylvestre* is the most distant taxonomic unit (De Bustos and Jouve, 2002). Analyses of RFLP, AFLP, SSR, and ISSR also indicated that *S. sylvestre* is one of the most ancient species (Chikmawati et al., 2005; Shang et al., 2006; Skuza et al., 2007; Ren et al., 2011). The analysis of noncoding regions of the cpDNA and mtDNA also indicated the divergence of *S. sylvestre* from other species and subspecies of rye (Skuza et al., 2019). ND-FISH results of (AAC)_6_ showed different signal patterns between *S. sylvestre* and other rye varieties (He et al., 2021). In this study, the signal patterns of (AAC)_6_, Oligo-pSc119.2-1, Oligo-pTa71A-2, Oligo-pSc200 + Oligo-pSc250 showed different signal patterns between *S. Sylvestre* and other 14 rye varieties (**Figures 1**, **4**, **6**). The signal patterns of Oligo-pSc200 + Oligo-pSc250 on 7 chromosomes of *S. sylvestre* were completely different from other rye varieties (**Figure 4**). And the results of the cluster analysis also showed that *S. sylvestre* was highly divergent from other rye varieties, which was consistent with the results of previous studies (Ren et al., 2011; Tang et al., 2011; Cuadrado and Jouve, 2002).

Several studies proposed that cultivated rye had been domesticated from weedy rye, rather than directly from wild species (Sun et al., 2022). The RFLP analysis of mtDNA showed that *S. cereale* subsp. *Segetale* had a closer genetic relationship with *S. sylvestre* among the taxa (Skuza et al., 2007). The ISSR analysis also indicated greater similarity among the weedy subspecies than among different varieties of cultivated rye subspecies (Ren et al., 2011), which meant the leave of genetic differentiation among weedy rye was smaller than that of cultivated rye varieties. In this study, lower polymorphisms in *S. cereale* subsp. *Segetale* were observed (**Figure 7**). However, another weedy rye *S. cereale* subsp. *Dighoricum* showed higher chromosome differentiation than that of *S. cereale* subsp. *Segetale* (**Figure 7**). Sun et al. (2022) indicated that interspecific introgression serves as one of the likely causes of obscure species taxonomy of rye. In this study, the cluster analysis based on FISH signal patterns of cultivated rye, weedy rye, and wild rye (except *S. sylvestre)* also showed the same conclusion as Sun et al. (2022). All results from this study confirmed chromosome differentiation and high levels of chromosome diversity in different rye varieties.

## 5 Conclusion

In this study, 15 rye varieties including *S. cereale* (9 cultivated rye and 2 weedy rye), *S. strictum* (wild rye), *S. sylvestre* (wild rye), and *S. vavilovii* (wild rye) were examined by oligonucleotides probes. 30 signal sites and 748 signal patterns, 39 signal sites, and 816 signal patterns, 22 signal sites and 480 signal patterns, 1 signal site and 30 signal patterns were detected by Oligo-pSc119.2-1, (AAC)_6_, Oligo-pSc200 + Oligo-pSc250, and Oligo-pTa71A-2, respectively. The results indicated that high polymorphism occurs in the genomes of different rye species. The degree of variation of signal patterns on different chromosomes was not consistent, indicating that different chromosomes have different degrees of evolutionary conservation. 1R, 6R, and 7R have the highest variation rate and the fastest evolution, while 2R and 3R have the least variation rate and tend to be conservative in evolution. Moreover, the cluster analysis also indicated that *S. sylvestre* has the most far genetic distance from other rye species, and the ancestor of Chinese rye varieties may be *S. vavilovii*. This also suggested that these undomesticated and unselected Chinese rye varieties may contain more favorable genes in the genome, which may provide more genetic resources for distant hybridization breeding of wheat in the future.

## Data Availability

The datasets presented in this study can be found in online repositories. The names of the repository/repositories and accession number(s) can be found in the article/**Supplementary Material**.
