# Supplementary material for: Frequent variations and phylogenetic relationships within the genus Secale identified by ND-FISH according to the genome-wide universal oligonucleotides chromosome probes
Source: Front Plant Sci. 2024 Dec 12;15:1501642. doi: 10.3389/fpls.2024.1501642 (PMC11669505; doi:10.3389/fpls.2024.1501642)
Supplement: Supplementary file 1 [file Table1.docx]

Table S1 Oligonucleotide probe sequences used in this study

| Probes | sequences（5’-3’） |
| --- | --- |
| (AAC)_6_ | AACAACAACAACAACAAC |
| Oligo-pTa71A-2 | CCGACGGCCGTCGTGGACGGAAGTTGACGCGCGCCATGGAAAACTG |
| Oligo-pSc119.2-1 | CCGTTTTGTGGACTATTACTCACCGCTTTGGGGTCCCATAGCTAT |
| Oligo-pTa535-1 | AAAAACTTGACGCACGTCACGTACAAATTGGACAAACTCTTTCGGAGTATCAGGGTTTC |
| Oligo-Ku | GATCGAGACTTCTAGCAATAGGCAAAAATAGTAATGGTATCCGGGTTCG |
| Oligo-pSc200 | CTCACTTGCTTTGAGAGTCTCGATCAATTCGGACTCTAGGTTGATTTTTGTATTTTCT |
| Oligo-pSc250 | TGTGTTGTTCTTGGACAAAACAATGCATACCATCTCTTCTAC |
