# Supplementary material for: Frequent variations and phylogenetic relationships within the genus Secale identified by ND-FISH according to the genome-wide universal oligonucleotides chromosome probes
Source: Front Plant Sci. 2024 Dec 12;15:1501642. doi: 10.3389/fpls.2024.1501642 (PMC11669505; doi:10.3389/fpls.2024.1501642)
Supplement: Supplementary file 4 [file Table4.docx]

Table S4 Variation rates of all probes at different loci compared with Weining rye. The red spots are the probes and loci with a mutation rate of more than 60%

| Probe and locus | The mutation rate |
| --- | --- |
| Oligo-pTa71A-2 | 66.67% |
| Oligo-pSc200+Oligo-pSc250(1RS) | 53.33% |
| Oligo-pSc200+Oligo-pSc250(1RL) | 56.67% |
| Oligo-pSc200+Oligo-pSc250(2RS) | 40% |
| Oligo-pSc200+Oligo-pSc250(2RL-1) | 40% |
| Oligo-pSc200+Oligo-pSc250(2RL-2) | 80% |
| Oligo-pSc200+Oligo-pSc250(3RS) | 23.33% |
| Oligo-pSc200+Oligo-pSc250(3RL) | 56.67% |
| Oligo-pSc200+Oligo-pSc250(4RS) | 76.67% |
| Oligo-pSc200+Oligo-pSc250(4RL-1) | 70% |
| Oligo-pSc200+Oligo-pSc250(4RL-2) | 20% |
| Oligo-pSc200+Oligo-pSc250(5RS) | 60% |
| Oligo-pSc200+Oligo-pSc250(5RL-1) | 40% |
| Oligo-pSc200+Oligo-pSc250(5RL-2) | 26.67% |
| Oligo-pSc200+Oligo-pSc250(6RS-1) | 76.67% |
| Oligo-pSc200+Oligo-pSc250(6RS-2) | 10% |
| Oligo-pSc200+Oligo-pSc250(6RL-1) | 60% |
| Oligo-pSc200+Oligo-pSc250(6RL-2) | 50% |
| Oligo-pSc200+Oligo-pSc250(6RL-3) | 83.33% |
| Oligo-pSc200+Oligo-pSc250(7RS-1) | 36.67% |
| Oligo-pSc200+Oligo-pSc250(7RS-2) | 3.33% |
| Oligo-pSc200+Oligo-pSc250(7RL-1) | 76.67% |
| Oligo-pSc200+Oligo-pSc250(7RL-2) | 63.33% |
| Oligo-pSc119.2-1(1RS-1) | 16.67% |
| Oligo-pSc119.2-1(1RS-2) | 20% |
| Oligo-pSc119.2-1(1RL-1) | 53.33% |
| Oligo-pSc119.2-1(1RL-2) | 16.67% |
| Oligo-pSc119.2-1(1RL-3) | 36.67% |
| Oligo-pSc119.2-1(2RS) | 6.67% |
| Oligo-pSc119.2-1(2RL-1) | 6.67% |
| Oligo-pSc119.2-1(2RL-2) | 26.67% |
| Oligo-pSc119.2-1(2RL-3) | 40% |
| Oligo-pSc119.2-1(3RS) | 60% |
| Oligo-pSc119.2-1(3RL-1) | 6.67% |
| Oligo-pSc119.2-1(3RL-2) | 53.33% |
| Oligo-pSc119.2-1(4RS) | 40% |
| Oligo-pSc119.2-1(4RL-1) | 76.67% |
| Oligo-pSc119.2-1(4RL-2) | 23.33% |
| Oligo-pSc119.2-1(4RL-3) | 40% |
| Oligo-pSc119.2-1(5RS-1) | 13.33% |
| Oligo-pSc119.2-1(5RS-2) | 33.33% |
| Oligo-pSc119.2-1(5RL-1) | 26.67% |
| Oligo-pSc119.2-1(5RL-2) | 30% |
| Oligo-pSc119.2-1(6RS-1) | 53.33% |
| Oligo-pSc119.2-1(6RS-2) | 56.67% |
| Oligo-pSc119.2-1(6RL-1) | 50% |
| Oligo-pSc119.2-1(6RL-2) | 30% |
| Oligo-pSc119.2-1(6RL-3) | 53.33% |
| Oligo-pSc119.2-1(6RL-4) | 46.67% |
| Oligo-pSc119.2-1(7RS) | 26.67% |
| Oligo-pSc119.2-1(7RL-1) | 50% |
| Oligo-pSc119.2-1(7RL-2) | 73.33% |
| Oligo-pSc119.2-1(7RL-3) | 26.67% |
| (AAC)6(1R-1) | 43.33% |
| (AAC)6(1R-2) | 30% |
| (AAC)6(1R-3) | 20% |
| (AAC)6(1R-4) | 36.67% |
| (AAC)6(1R-5) | 6.67% |
| (AAC)6(1R-6) | 40% |
| (AAC)6(2R-1) | 6.67% |
| (AAC)6(2R-2) | 33.33% |
| (AAC)6(2R-3) | 26.67% |
| (AAC)6(2R-4) | 33.33% |
| (AAC)6(2R-5) | 0% |
| (AAC)6(2R-6) | 30% |
| (AAC)6(3R-1) | 16.67% |
| (AAC)6(3R-2) | 6.67% |
| (AAC)6(3R-3) | 26.67% |
| (AAC)6(3R-4) | 0% |
| (AAC)6(3R-5) | 0% |
| (AAC)6(3R-6) | 26.67% |
| (AAC)6(4R-1) | 6.66% |
| (AAC)6(4R-2) | 26.67% |
| (AAC)6(4R-3) | 20% |
| (AAC)6(4R-4) | 20% |
| (AAC)6(4R-5) | 6.67% |
| (AAC)6(4R-6) | 13.33% |
| (AAC)6(5R-1) | 30% |
| (AAC)6(5R-2) | 23.33% |
| (AAC)6(5R-3) | 33.33% |
| (AAC)6(5R-4) | 70% |
| (AAC)6(5R-5) | 76.67% |
| (AAC)6(5R-6) | 70% |
| (AAC)6(5R-7) | 50% |
| (AAC)6(5R-8) | 6.67% |
| (AAC)6(6R-1) | 63.33% |
| (AAC)6(6R-2) | 30% |
| (AAC)6(6R-3) | 10% |
| (AAC)6(6R-4) | 33.33% |
| (AAC)6(7R-1) | 56.67% |
| (AAC)6(7R-2) | 10% |
| (AAC)6(7R-3) | 60% |
